# The Prevalence and Psychopathological Correlates of Sibling Bullying in Children with and without Autism Spectrum Disorder

**DOI:** 10.1007/s10803-018-3484-2

**Published:** 2018-02-08

**Authors:** Umar Toseeb, Gillian McChesney, Dieter Wolke

**Affiliations:** 1 Department of Education, Derwent College, University of York, York, YO10 5DD UK; 2 Department of Psychology, Manchester Metropolitan University, Brooks Building, 53 Bonsall Street, Manchester, M15 6GX UK; 3 Department of Psychology, University of Warwick, Coventry, CV4 7AL UK

**Keywords:** Sibling bullying, Autism spectrum disorder, Psychopathology, Childhood, Prosocial, Millennium Cohort Study

## Abstract

Using data from a prospective population based study, the prevalence and psychopathological correlates of sibling bullying in children with and without autism spectrum disorder (ASD) were estimated. There were 475 children with ASD and 13,702 children without ASD aged 11 years. Children with ASD were more likely to be bullied by their siblings compared to those without ASD. They were also more likely than those without ASD to both bully and be bullied by their siblings, which was associated with lower prosocial skills as well as more internalizing and externalizing problems compared to those not involved in any sibling bullying. Interventions to improve social and emotional outcomes in children with ASD should focus on both the affected and the unaffected sibling.

## Introduction

Autism spectrum disorder (ASD) is characterized by social and communication difficulties, repetitive behaviours, and high sensitivity to sensory stimulus (American Psychiatric Association 2013). In the UK, the prevalence of ASD has been estimated at 1–3% (Baird et al. 2006; Dillenburger et al. 2015). ASD has a number of psychopathological correlates, which further reduce the quality of life in affected individuals (Matson and Nebel-Schwalm 2007; van Steensel et al. 2011). Children with ASD have difficulties in social interactions, such as turn taking in conversation, and deficits in non-verbal communication (American Psychiatric Association 2013). These difficulties have implications for childrens’ relationships with the people around them.

In the UK, 85% of children have at least one sibling. Good quality sibling relationships are important as they help children to develop social skills and are a source of emotional support (Brown et al. 1996; Downey and Condron 2004; Stormshak et al. 1996). However, sibling relationships can also include frequent conflict and aggression. Up to 50% of children have been bullied by their siblings and up to 40% have bullied their siblings (Wolke et al. 2015). Sibling bullying is defined as “any unwanted aggressive behaviour(s) by a sibling that involves an observed or perceived power imbalance and is repeated multiple times or is highly likely to be repeated; bullying may inflict harm or distress on the targeted sibling, including physical, psychological, or social harm. It encompasses two modes of bullying (direct and indirect) as well as four types of bullying (physical, verbal, relational, and damage to property)” (Wolke et al. 2015, p. 918). Boys are more likely to bully their siblings (Tippett and Wolke 2015) and girls and younger children are more likely to be victims of sibling bullying, usually by an older sibling (Bowes et al. 2014). As the number of children in the household increases, so does the rate of sibling bullying (Bowes et al. 2014; Tippett and Wolke 2015).

Sibling bullying is associated with higher levels of depression and loneliness (Duncan 1999), more behavioural problems (Wolke and Samara 2004; Wolke and Skew 2011), and higher levels of mental distress (Tucker et al. 2014b). Furthermore, children who are bullied by a sibling at the age of 12 years old are more likely to have depression, anxiety, and have self-harmed by the age of 18 years old compared to those who are not (Bowes et al. 2014).

There are different roles siblings can take in their involvement in bullying. The ‘bully only’ children bully their siblings but are not bullied themselves. The ‘victim only’ children are bullied but they do not bully their siblings. ‘Bully-victims’ are children who are perpetrators of sibling bullying and also become victims at other times. Involvement in these different sibling bullying roles is associated with different levels of psychopathology. Bully-victims who engage in physical and relational bullying have more social-emotional difficulties compared to victim only and bully only children (Wolke and Samara 2004). Moreover, bully-victims who engage in relational bullying are less prosocial compared to the other two types (Wolke and Samara 2004).

To the best of our knowledge, there are no reports on the prevalence of sibling bullying in children with ASD. It is, however, well documented that peer bullying in children with ASD is higher compared to the children without ASD (Cappadocia et al. 2012; Little 2002; Sterzinget al. 2012; van Roekel et al. 2010). This heightened risk of being bullied by peers in children with ASD may be mirrored in vulnerability to sibling bullying (Hebron et al. 2015).

One of the reasons why children with ASD may be at increased risk for sibling bullying is due to their social and communication difficulties, which are related to peer bullying in children with ASD (Cappadocia et al. 2012). Conversely, good are a protective factor in peer bullying (Cowie and Sharp 1994). Secondly, sibling bullying may be more likely in families who have a child with ASD due to a higher risk of poorer communication skills within these families. There is evidence for social impairment (Constantino et al. 2006), language difficulties (Toth et al. 2007), and worse social-communicative interactions (Stoner et al. 2007) in siblings of children with ASD. This also extends to parents of children with ASD (Dawson et al. 2007). Therefore, the broader autism phenotype in family members might make undiagnosed siblings more likely to bully and subsequently it may exacerbate social difficulties experienced by children with ASD. Such suboptimal levels of communication for siblings may lead to higher levels of sibling bullying in families who have a child with ASD compared to the general population. Thirdly, siblings of children with ASD have been reported to have more behavioural problems compared to their typically developing peers (Bagenholm and Gillberg 1991; Verte et al. 2003), which may increase the potential for conflict between siblings. Finally, reactive aggression is higher in boys with ASD compared to those without ASD (Kaartinen et al. 2014), which may also be associated with more sibling bullying. Indeed, when unaffected siblings were asked to identify a recent problem with their affected sibling, over half reported aggressive behaviour (Ross and Cuskelly 2006). It is, however, unclear how this compares to sibling dyads in which neither child has ASD, as there was no control sample. There is a range of evidence why one might expect more sibling bullying experiences in children with ASD. On the other hand, there are also reports that siblings of children with ASD report less conflict with their affected sibling compared to typically developing children (Kaminsky and Dewey 2001) and studies that point towards positive relationships, or at least no adverse relationships (see Meadan et al. 2010 for a review). Such mixed findings support the case for further research on the sibling interactions where one child has ASD.

In this prospective longitudinal study, we investigated sibling bullying, and the associated psychopathological adversities, in children with and without ASD. We hypothesized that children with ASD (child has ASD but their sibling does not) would experience higher levels of sibling bullying compared to those without ASD (child and sibling do not have ASD). The psychopathological correlates of sibling bullying were also investigated. In line with previous work, we expected to find that those children who are involved in sibling bullying will have worse psychopathology compared to those who are not involved in any sibling bullying.

## Method

### Study Sample

The Millennium Cohort Study (MCS) is a longitudinal cohort of children drawn from all live births in the United Kingdom between 2000 and 2002. Electoral wards were used to randomly select the MCS sample population, ensuring that all four areas of the UK (England, Scotland, Wales, Northern Ireland) were adequately represented. Ethnic minorities and deprived areas were oversampled using disproportionate stratification. Primary caregivers, usually a parent, and the child contributed to data collection. Data was accessed via the UK Data Archive (http://www.data-archive.ac.uk/). Further details of the MCS cohort are reported elsewhere (Connelly and Platt 2014). In this study, data collected during wave 5 (age 11 years) were analysed. Covariates from earlier waves were also used (psychopathology when the child was 3 years old and harsh parenting when the child 7 years old). Twins and those who did not have any siblings were excluded. Therefore, only one child per family was included the analysis. As described in the subsequent paragraphs, each child was assigned to only one of two mutually exclusive groups (with ASD or without ASD). Data was collected from parents and one child but not the siblings. Therefore, no information about the siblings, such as ASD diagnosis status, was available to include in the analysis. The total weighted sample size was N: 14,177 (unweighted 11,687).

#### Children with Autism Spectrum Disorder

When the child was aged 5, 7, and 11 years, the primary caregiver was asked, “Has a doctor or health professional ever told you that [child] had Autism, Asperger’s syndrome or autistic spectrum disorder?”. Those children whose primary care givers responded positively to the question at any one of the three time points were identified as children with ASD. There were 475 children (81% Boys) with ASD (mean age 10.66 years, 95% CI 10.59–10.72).

#### Children without Autism Spectrum Disorder

The remainder of the sample was used as a comparison group, which will be referred to as children without ASD. There were 13,702 children (51% Boys) without ASD (mean age 10.67 years, 95% CI 10.65–10.68).

### Measures

#### Sibling Bullying

When the child was aged 11 years, he/she was asked to respond to two questions on a six-point scale (never, less often, every few months, approximately once a month, approximately once a week, most days): “how often do your brothers or sisters hurt you or pick on you on purpose?” (victimization) and “how often do you hurt or pick on your brothers or sisters on purpose?” (perpetration). Based on previous work (Wolke and Samara 2004), mutually exclusive sibling bullying groups were then defined as follows; victim only: victimised at least once a week but not perpetrated at least once a week; bully only: perpetrated at least once a week but not victimised at least once a week; bully-victim: both perpetrated and victimized at least and once a week.

### Socio-demographic Data

Primary caregivers were asked to choose their child’s ethnicity from a list of options. A dummy variable was created (non-White or White). They were also asked to list income from all sources (e.g. main job, government benefits etc.), which was used to calculate their overall income. This was standardised using the OECD-modified scale (Hagenaars et al. 1994). Those families who were below the 60% median income level were categorised as low household income. Primary caregivers completed a grid about other members of the household. This was used to determine lone parent status (one parent/carer or two parents/carers), number of siblings (1, 2, 3, 4 or more), and birth order (1st, 2nd, 3rd, 4th or later).

#### Psychopathology

The primary caregiver completed Strengths and Difficulties Questionnaire, (SDQ, Goodman 1997) when the child was aged 3 years (covariate) and 11 years (outcome). As per the instructions for scoring, the emotional and peer problems subscales were summed to create a measure of internalizing symptoms (0–20). Conduct and hyperactivity subscales were summed to create a measure of externalizing symptoms (0–20). The prosocial subscale was used to measure prosocial skills (0–10). Higher scores indicated more internalizing symptoms, more externalizing symptoms, and better prosocial skills. The internal reliability for all three measures was high (internalizing 0.98, externalizing 0.98, prosocial skills 0.99). The SDQ has previously been used to assess psychopathology in children with developmental disorders such as ASD and Developmental Language Disorder (Baird et al. 2006; Pickles et al. 2016; Russell et al. 2013; Toseeb et al. 2017)

#### Harsh Parenting

When the child was aged 7 years, primary caregivers were asked about how often they smack, shout, or tell their child off on a five-point scale (never, rarely, sometimes, often, daily). Higher summed scores indicated harsher parenting. This was used as a covariate in statistical models.

#### Cognitive Measures

At age 11 years, the verbal similarities subscale of the British Ability Scales (Elliot et al. 1996) was used to assess the child’s verbal ability. Scoring instructions were used to generate standardised scores. Higher scores indicated better verbal ability. The Cambridge Neuropsychological Test Automated Battery (CANTAB) Spatial Working Memory Task (Robbins et al. 1994) was used as a proxy for cognitive function. The total number of errors was used and reverse scored so that a higher score indicated better cognitive function. Both measures were used as indicative of wider cognitive function in the absence of a full battery of cognitive data being available.

### Statistical Analyses

All analyses were conducted using Stata/SE 14.2 (StataCorp 2015) and two tailed tests, p < .05, were used. To account for unequal sample attrition and the application of disproportionate stratification, all estimates were weighted to population level. All reported values are weighted estimates.

Two multivariable ordered logistic regression models were run to calculate odds ratios for the prevalence of sibling bullying (one for sibling bullying victimization, Model 1 in Table 2, and the other sibling bullying perpetration, Model 2 in Table 2). The predictors were entered as ASD group, gender, ethnicity, household income, lone parent status, number of siblings, birth order, harsh parenting score, verbal ability, and cognitive function. Then, relative risk ratios were calculated using logistic regression models to investigate membership of sibling bullying involvement groups (the prevalence of groups in shown in Table 3 and relative risk ratios in Table 4). The outcome variable was entered as the sibling bullying group (neither, victim only, bully only, bully-victim). In separate models the predictors were entered as ASD group, gender, ethnicity, household income, lone parent status, number of siblings, harsh parenting score, birth order, verbal ability, and cognitive function. The model for ASD group was repeated with (1) gender, ethnicity, household income, lone parent status, number of siblings, harsh parenting score, and birth order as covariates and (2) with all of the covariates previously specified and additionally verbal ability and cognitive function.

Psychopathological correlates of sibling bullying were estimated using three linear regression models (Table 5). Collinearity tests indicated that the data met the assumption of multicollinearity (tolerance 0.72–0.98 and VIF 1.02–1.36). The outcome variable was either internalizing symptoms, externalizing symptoms, or prosocial skills. The predictors were bullying group (neither, victim only, bully only, or bully-victim), which were entered as a dummy variable, ASD group, bullying group x ASD group interaction, gender, household income, ethnicity, lone parent status, psychopathology at age 3 years, number of siblings, birth order, harsh parenting score, verbal ability, and cognitive function.

## Results

### Prevalence of Sibling Bullying

Descriptive statistics for the prevalence of sibling bullying are reported in Table 1.

**Table 1:** Prevalence of sibling bullying

	Any victimization							Any perpetration						
	Total N	Never	Less often	Every few months	~ Once a month	~ Once a week	Most days	Total N	Never	Less often	Every few months	~ Once a month	~ Once a week	Most days
Overall	13597 (100%)	2940 (22%)	2800 (20%)	808 (6%)	998 (7%)	2841 (21%)	3210 (24%)	13644 (100%)	3387 (25%)	3557 (26%)	997 (7%)	1224 (9%)	2722 (20%)	1757 (13%)
ASD status														
Without ASD	13189 (100%)	2868 (22%)	2740 (21%)	777 (6%)	970 (7%)	2762 (21%)	3072 (23%)	13231 (100%)	3282 (25%)	3475 (26%)	959 (7%)	1197 (9%)	2635 (20%)	1682 (13%)
With ASD	408 (100%)	72 (18%)	60 (15%)	31 (7%)	27 (7%)	80 (19%)	138 (34%)	413 (100%)	105 (25%)	82 (20%)	38 (9%)	27 (7%)	86 (21%)	75 (18%)
Gender														
Boys	6975 (100%)	1611 (23%)	1403 (20%)	392 (6%)	524 (8%)	1416 (20%)	1629 (23%)	7024 (100%)	1773 (25%)	1734 (25%)	541 (8%)	662 (9%)	1389 (20%)	924 (13%)
Girls	6622 (100%)	1329 (20%)	1397 (21%)	416 (6%)	473 (7%)	1425 (22%)	1581 (24%)	6621 (100%)	1614 (24%)	1823 (28%)	456 (7%)	562 (8%)	1332 (20%)	833 (13%)
Ethnicity														
Non-white	2104 (100%)	617 (29%)	503 (24%)	113 (5%)	149 (7%)	293 (14%)	429 (21%)	2107 (100%)	660 (31%)	616 (29%)	116 (5%)	127 (7%)	334 (16%)	253 (12%)
White	11490 (100%)	2321 (20%)	2297 (20%)	695 (6%)	848 (8%)	2548 (22%)	2780 (24%)	11534 (100%)	2725 (24%)	2940 (25%)	881 (8%)	1097 (9%)	2388 (21%)	1504 (13%)
Household income														
High	10459 (100%)	2151 (21%)	2123 (20%)	655 (6%)	857 (8%)	2351 (23%)	2322 (22%)	10496 (100%)	2476 (23%)	2700 (26%)	817 (8%)	1060 (10%)	2200 (21%)	1242 (12%)
Low	3138 (100%)	789 (25%)	677 (21%)	153 (5%)	141 (5%)	490 (16%)	888 (28%)	3149 (100%)	911 (29%)	857 (27%)	180 (6%)	164 (5%)	522 (17%)	515 (16%)
Lone parent														
No	10369 (100%)	2202 (21%)	2186 (21%)	624 (6%)	786 (8%)	2250 (22%)	2322 (22%)	10411 (100%)	2491 (24%)	2746 (26%)	772 (7%)	997 (10%)	2134 (21%)	1270 (12%)
Yes	3228 (100%)	738 (23%)	614 (19%)	184 (6%)	212 (7%)	591 (18%)	888 (27%)	3234 (100%)	895 (28%)	811 (25%)	225 (7%)	227 (7%)	588 (18%)	487 (15%)
Number of siblings														
1	6642 (100%)	1599 (24%)	1351 (21%)	415 (6%)	484 (7%)	1397 (21%)	1395 (21%)	6685 (100%)	1785 (27%)	1673 (25%)	478 (7%)	620 (9%)	1304 (20%)	825 (12%)
2	4235 (100%)	817 (19%)	856 (20%)	247 (6%)	331 (8%)	948 (22%)	1035 (25%)	4231 (100%)	926 (22%)	1155 (27%)	322 (8%)	410 (10%)	909 (21%)	509 (12%)
3	1834 (100%)	356 (19%)	389 (21%)	95 (5%)	123 (7%)	354 (20%)	517 (28%)	1840 (100%)	445 (24%)	489 (27%)	138 (7%)	140 (8%)	352 (19%)	277 (15%)
4 or more	886 (100%)	167 (19%)	204 (23%)	51 (6%)	59 (6%)	141 (16%)	263 (30%)	888 (100%)	231 (26%)	240 (27%)	58 (7%)	55 (6%)	157 (18%)	146 (16%)
Birth order														
1st	4790 (100%)	1187 (25%)	913 (19%)	252 (5%)	321 (7%)	983 (21%)	1132 (23%)	4821 (100%)	1010 (21%)	1206 (25%)	335 (7%)	473 (10%)	1040 (21%)	758 (16%)
2nd	5043 (100%)	868 (17%)	1100 (22%)	342 (7%)	391 (8%)	1194 (23%)	1148 (23%)	5055 (100%)	1199 (24%)	1385 (27%)	405 (8%)	441 (9%)	1087 (22%)	538 (11%)
3rd	2088 (100%)	488 (23%)	451 (22%)	114 (6%)	147 (7%)	379 (18%)	508 (24%)	2093 (100%)	633 (30%)	578 (28%)	134 (6%)	162 (8%)	361 (17%)	225 (11%)
4th or later	1077 (100%)	239 (22%)	217 (20%)	68 (6%)	94 (9%)	186 (17%)	273 (26%)	1075 (100%)	363 (34%)	256 (24%)	86 (8%)	84 (8%)	133 (12%)	153 (14%)

The logistic regression model for sibling bullying victimization (Model 1 in Table 2) showed that having ASD, being a girl, of White ethnicity, having more siblings, and experiencing harsher parenting were all associated with increased odds of being bullied by a sibling. The logistic regression model for sibling bullying perpetration (Model 2 in Table 2) showed that being of White ethnicity, having more siblings, harsher parenting, and better cognitive function were associated with increased odds of bullying a sibling. Being from a low-income family and being a younger sibling was associated with decreased odds of bullying a sibling.

**Table 2:** Multivariable ordered logistic regression models

	OR [95% CI]	p value
Model 1: sibling bully victimization		
With ASD	1.45[1.15, 1.85]	.002
Girls	1.11 [1.03, 1.20]	.005
White	1.41 [1.19, 1.66]	< .001
Low-income	0.88 [0.75, 1.04]	.141
Lone parent family	1.12 [0.99, 1.27]	.066
Number of siblings	1.19 [1.12, 1.27]	< .001
Birth order	0.98 [0.93, 1.03]	.407
Harsh parenting	1.12 [1.09, 1.14]	< .001
Verbal ability	1.00 [0.99, 1.00]	.062
Cognitive function	1.00 [1.00, 1.00]	.851
Model 2: sibling bully perpetration		
With ASD	1.20[0.94, 1.54]	.138
Girls	1.01 [0.93, 1.10]	.724
White	1.34 [1.14, 1.58]	.001
Low-income	0.85 [0.72,0.99]	.041
Lone parent family	1.02 [0.90, 1.14]	.785
Number of siblings	1.22 [1.15, 1.30]	< .001
Birth order	0.78 [0.74, 0.82]	< .001
Harsh parenting	1.14 [1.12, 1.17]	< .001
Verbal ability	1.00 [0.99, 1.00]	.285
Cognitive function	1.00[1.00, 1.01]	.026

*OR* odds ratio, *95% CI* 95% confidence intervals

### Types of Sibling Bullying Involvement

Based on their responses to the sibling bullying involvement questions, each child was categorised into one of the mutually exclusive sibling bullying groups. The prevalence of these groups is shown in Table 3 and the results of the multinomial logistic regressions are shown in Table 4.

**Table 3:** Prevalence of types and predictors of sibling bullying involvement

	Distribution	Neither *n* (%)	Victim only *n* (%)	Bully only *n* (%)	Bully-victim *n* (%)
Overall	13519 (100%)	6925 (52%)	2160 (16%)	577 (5%)	3858 (27%)
ASD status					
Without ASD	13112 (100%)	6753 (50%)	2084 (16%)	560 (5%)	3716 (29%)
With ASD	407 (100%)	172 (42%)	77 (19%)	17 (4%)	141 (35%)
Gender					
Boys	6940 (100%)	3522 (51%)	1134 (16%)	391 (6%)	1894 (27%)
Girls	6579 (100%)	3403 (52%)	1026 (15%)	186 (3%)	1964 (30%)
Ethnicity					
Non-white	2090 (100%)	1271 (61%)	241 (11%)	102 (5%)	477 (23%)
White	11426 (100%)	5652 (49%)	1918 (17%)	475 (4%)	3381 (30%)
Household income					
High	10400 (100%)	5362 (51%)	1633 (16%)	395 (4%)	3011 (29%)
Low	3119 (100%)	1563 (50%)	528 (17%)	182 (6%)	847 (27%)
Lone parent					
No	10314 (100%)	5337 (52%)	1607 (16%)	429 (4%)	2941 (28%)
Yes	3205 (100%)	1588 (49%)	554 (17%)	147 (5%)	916 (29%)
Number of siblings					
1	6607 (100%)	3578 (54%)	931 (14%)	252 (4%)	1847 (28%)
2	4206 (100%)	2034 (48%)	761 (18%)	203 (5%)	1209 (29%)
3	1824 (100%)	873 (48%)	328 (18%)	84 (5%)	539 (29%)
4 or more	882 (100%)	440 (50%)	141 (16%)	38 (4%)	263 (30%)
Birth order					
1st	4770 (100%)	2355 (49%)	633 (13%)	306 (7%)	1475 (31%)
2nd	5019 (100%)	2538 (51%)	867 (17%)	153 (3%)	1461 (29%)
3rd	2067 (100%)	1129 (55%)	362 (17%)	60 (3%)	516 (25%)
4th or later	1068 (100%)	581 (54%)	204 (19%)	31 (3%)	251 (24%)

**Table 4:** Logistic regression analyses sibling bullying involvement groups

	Victim only		Bully only		Bully-victim	
	RRR [95% CI]	p Value	RRR [95% CI]	p Value	RRR [95% CI]	p Value
With ASD^a^	1.44 [0.98, 2.13]	.064	1.19 [0.56, 2.52]	.644	1.49 [1.09, 2.04]	.013
Girls	0.94 [0.82, 1.06]	.316	0.49 [0.39, 0.63]	< .001	1.07 [0.97, 1.19]	.183
White	1.78 [1.49, 2.13]	< .001	1.05 [0.79, 1.40]	.724	1.59 [1.32, 1.93]	< .001
Low-income	1.11 [0.92, 1.33]	.274	1.58 [1.21, 2.18]	.001	0.96 [0.84, 1.11]	.620
Lone parent	1.16 [0.99, 1.36]	.070	1.15 [0.87, 1.52]	.311	1.05 [0.92, 1.19]	.492
2 siblings	1.44 [1.23, 1.67]	< .001	1.42 [1.11, 1.81]	.006	1.15 [1.03, 1.29]	.018
3 siblings	1.44 [1.16, 1.79]	.001	1.37 [0.98, 1.91]	.061	1.20 [1.01, 1.42]	.041
4 or more siblings	1.23 [0.91, 1.66]	.178	1.21 [0.72, 2.06]	.473	1.16 [0.91, 1.48]	.234
Harsh parenting	1.06 [1.02, 1.10]	.001	1.11 [1.09, 1.27]	< .001	1.17 [1.13, 1.20]	< .001
2nd born	1.27 [1.08, 1.49]	.004	0.46 [0.35, 0.61]	< .001	0.92 [0.82, 1.03]	.161
3rd born	1.19 [0.98, 1.46]	.086	0.41 [0.28, 0.60]	< .001	0.73 [0.62, 0.86]	< .001
4th or later born	1.31 [1.01, 1.69]	.040	0.41 [0.27, 0.64]	< .001	0.69 [0.55, 0.86]	.001
Verbal ability	1.00 [0.99, 1.00]	.056	0.99 [0.99, 1.00]	.053	0.99 [0.99, 0.99]	.004
Cognitive function	1.00 [0.99, 1.00]	.115	1.00 [0.99, 1.00]	.584	1.00 [1.00, 1.00]	.588

*RRR* relative risk ratio, *95% CI* 95% confidence intervals. Base outcome is the Neither group

^a^Model for bully-victim remained significant when gender, ethnicity, household income, lone parent status, number of siblings, harsh parenting score, and birth order were included as covariates (1.40[1.01, 1.93], p = .040) but not when verbal ability and cognitive function was also included (1.37 [0.98, 1.91], p = .068).

Groups were compared to the neither group based on the variables of interest. Children with ASD were more likely than those without ASD to be in the bully-victim group, and this effect remained after controlling for socio-demographic variables but not when also controlling for verbal ability and cognitive function (see note on Table 4). Girls were less likely than boys to be in the bully only group. Children from a White ethnic background were more likely than children from a non-White ethnic background to be in the victim only group and bully-victim group. Those from a low-income household were more likely than those from a high-income household be in the bully only group. Those with two or three siblings were more likely than those with one sibling to be in the victim only and bully-victim groups. The children with only two siblings were more likely than those with one to be in the bully only group. Children of parents who adopted harsher parenting were more likely to be in the victim only, bully only, and bully-victim groups. For the most part, children who had more older siblings were more likely to be in the victim only group, less likely to be in the bully only group, and less likely to be in the bully-victim group (see Table 4 for exceptions).

### Psychopathological Correlates of Sibling Bullying

The results of linear regression analyses are shown in Table 5. When compared to the neither group, children in the victim only and bully-victim groups had more internalizing symptoms. Those in the bully-victim and bully only groups had more externalizing symptoms. There was a significant interaction between bullying involvement and ASD group for externalizing symptoms. Posthoc analysis showed that children with ASD in the victim only group had more externalizing symptoms but this was not the case for children without ASD. Having ASD was also predictive of higher internalizing and externalizing symptoms. That is, bullying involvement of any kind and having ASD were both independently predictive of externalizing psychopathologies even after gender, household income, ethnicity, lone parent status, earlier psychopathology, number of siblings, birth order, harsh parenting, verbal ability, and cognitive function were kept constant. This was also true for internalizing symptoms but only for victim only and bully-victim groups.

**Table 5:** Psychopathological correlates of types of sibling bullying

	Internalizing symptoms		Externalizing symptoms		Prosocial skills	
	Unstandardized Beta [95% CI]	p Value	Unstandardized Beta [95% CI]	p Value	Unstandardized Beta [95% CI]	p Value
Bullying involvement group						
Neither	0 [Reference]		0 [Reference]		0 [Reference]	
Victim only	0.44 [0.19, 0.69]	< .001	0.19 [− 0.01, 0.40]	.065	− 0.03 [− 0.15, 0.08]	.580
Bully only	0.15 [− 0.22, 0.51]	.421	0.62[0.23, 1.02]	.002	− 0.24[− 0.42, − 0.05]	.012
Bully-victim	0.34 [0.16, 0.52]	< .001	0.46[0.27, 0.65]	< .001	− 0.19 [− 0.27, − 0.11]	< .001
ASD group						
Without ASD	0 [Reference]		0 [Reference]		0 [Reference]	
With ASD	4.08 [3.20, 4.95]	< .001	2.65[1.78, 3.53]	< .001	− 1.30[− 1.83, − 0.78]	< .001
Bullying involvement group X ASD group						
Neither X ASD	0 [Reference]		0 [Reference]		0 [Reference]	
Victim only X ASD	1.19 [− 0.57, 3.01]	.184	1.63[0.29, 2.97]^+^	.017^a^	− 0.06 [− 0.95, 0.83]	.890
Bully only X ASD	− 0.99 [− 3.77, 1.78]	.483	0.03[− 2.94, 3.00]	.984	− 0.76 [− 2.81, 1.29]	.469
Bully-victim X ASD	0.47[− 0.95, 1.89]	.512	0.98[− 0.20, 2.15]	.102	− 0.06 [− 0.77, 0.66]	.873
Gender						
Boys	0 [Reference]		0 [Reference]		0 [Reference]	
Girls	0.26 [0.10, 0.42]	.001	− 0.73 [− 0.88, − 0.58]	< .001	0.41 [0.33, 0.50]	< .001
Household income						
High	0 [Reference]		0 [Reference]		0 [Reference]	
Low	0.49[0.21, 0.76]	< .001	0.72 [0.42, 1.03]	< .001	− 0.29 [− 0.44, − 0.16]	< .001
Ethnicity						
Non-white	0 [Reference]		0 [Reference]		0 [Reference]	
White	0.23 [− 0.07, 0.54]	.127	0.50[0.18, 0.82]	.002	− 0.06 [− 0.10, 0.22]	.453
Lone parent						
No	0 [Reference]		0 [Reference]		0 [Reference]	
Yes	0.43[0.22,0.64]	< .001	0.54[0.30, 0.78]	< .001	− 0.14 [− 0.27, − 0.02]	.021
Early psychopathology^a^	0.34[0.30,0.38]	< .001	0.31[0.29,0.34]	< .001	0.17 [0.15, 0.19]	< .001
Number of siblings	− 0.05[− 0.15,0.06]	.403	− 0.03[− 0.14,0.07]	.533	− 0.09 [− 0.09, − 0.03]	.006
Birth order	0.04 [− 0.06, 0.13]	.458	0.09 [− 0.00, 0.17]	.054	0.11 [0.05, 0.16]	< .001
Harsh parenting	0.16[0.12,0.20]	< .001	0.40[0.36,0.45]	< .001	− 0.12 [− 0.14, − 0.10]	< .001
Verbal ability	− 0.02 [− 0.03, − 0.01]	< .001	− 0.03 [− 0.04, − 0.02]	< .001	0.01 [0.00, 0.01]	.001
Cognitive function	− 0.01 [− 0.02, − 0.01]	< .001	− 0.02 [− 0.03, − 0.02]	< .001	0.00 [0.00, 0.00]	.035

^a^Psychopathological scores at age 3. For internalizing symptoms, early psychopathology refers to internalizing symptoms in the SDQ at age 3. For externalising symptoms, early psychopathology refers to externalizing symptoms in the SDQ at age 3. For prosocial skills, early psychopathology refers to prosocial scale of the SDQ at age 3

^+^Posthoc analyses showed that children with ASD in the victim only group had more externalizing symptoms (unstandardized beta 1.64 95% CI [0.09, 3.20], p = .039) but this was not the case for children without ASD (unstandardized beta 1.91 95% CI [− 0.12, 0.39], p = .065).

Similar to the internalizing and externalizing symptom findings, for prosocial skills, when compared to the neither group, children in the bully only and bully-victim groups were less prosocial. Having ASD was also predictive of lower prosocial skills compared to children without ASD. That is, bullying involvement in a perpetrator role (irrespective of being a victim or not) and having ASD were both independently predictive of lower prosocial skills when gender, household income, ethnicity, lone parent status, earlier psychopathology, number of siblings, birth order, harsh parenting, verbal ability, and cognitive function were held constant.

## Discussion

### Summary of Main Findings

In this population based prospective cohort study of children, it was found that children with ASD were more likely to be bullied by their siblings compared to those without ASD, in particular as bully-victim. This effect remained even after controlling for socio-demographic and family level variables and was associated with adverse psychopathologies.

These novel findings shed new light on the nature of sibling relationships in children with ASD. They are in line with previous research, which suggests elevated behaviour problems in children with ASD and their siblings (Bagenholm and Gillberg 1991; Sterzing et al. 2012; Verte et al. 2003). Our findings indicate that children with ASD are specifically at increased risk of sibling victimization as a bully-victim. This may indicate that children with ASD display reactive aggression in response to first being bullied by their siblings. In particular, boys with ASD have been found to more likely respond aggressively to mild forms of aggression (Kaartinen et al. 2014) and so it may be that such disproportionate responses lead to an escalation in sibling conflict. Alternatively, it may be that the aggression from the child with ASD is proactive and the non-affected sibling displays reactive aggression. In any case, these findings suggest that children with ASD are more likely to be involved in two-way bullying (bully-victim) rather than one way (victim only or bully only).

Apart from the novel finding that ASD is related to sibling victimisation, the study replicates previously reported findings of structural and parenting factors related to sibling bullying. As the number of siblings increases there is also an increase in the likelihood of both bullying perpetration and victimisation (Bowes et al. 2014; Tippett and Wolke 2015). In the current study, it was found that both victimization and perpetration was increased if parents were more likely to use harsh parenting approaches. This has been previously reported as the most consistent factor related to sibling bullying (Tippett and Wolke 2015; Wolke et al. 2015). In line with previous reports, White ethnicity (Tucker et al. 2013) and birth order (Bowes et al. 2014) were predictive of sibling bullying. Furthermore it has been reported by some (Tucker et al. 2014a) but not others (Wolke et al. 2015) that sibling bullying may be higher in lone parent compared to two parent households. In the current study, such an effect was not found. Overall, bullying, whether between peers or between siblings, has been conceptualised as being motivated by competition for resources. It may thus not be surprising that increases are found where there are more siblings. Studies on general sibling conflict (McHale et al. 1995; Volk et al. 2012) and on sibling bullying (Updegraff et al. 2005) have identified a link with differential parental treatment of siblings, suggesting that sibling bullying might be motivated by inequality and a desire to improve one’s status, thereby mimicking the motivations that underlie bullying at school. Given that children with ASD have more social and emotional difficulties, it may be that parents spend more time with the affected child and are more accommodating of their needs. The unaffected child may feel resentment at the perceived inequality and adopt bullying behaviour to improve his/her own status.

Consistent with previous findings (Bowes et al. 2014; Tucker et al. 2014b; Wolke and Skew 2011), an increase of both internalizing and externalizing problems in those involved in sibling bullying was found in this study. Furthermore, those involved in perpetrating sibling bullying, bullies and bully-victims, were less prosocial in their behaviour to peers. The adverse associations of sibling bullying on internalizing or externalizing problems were comparable to those living in a low-income household or being raised by a lone parent (Table 5).

Not surprisingly, considering the pervasive nature of ASD, the effects of ASD on internalizing, externalizing and lack of prosocial skills were all very large and by far larger than any of the other factors considered. However, being involved in sibling bullying further increased externalizing and internalizing problems in those with ASD. This suggests a double-dose effect of sibling bullying involvement and having ASD.

### Strengths, Limitations, and Implications

A major strength of the research reported here is the large population-based sample. This allowed for accurate estimates of sibling bullying in children with and without ASD. Studies of clinical populations suffer from issues such as referral bias, which may lead to inaccurate estimates. Additionally, the large battery of data that was collected from each family allowed for a number of covariates to be included in all statistical models. This ensured that the effects that were observed were unique to the variables of interest. Despite best efforts, residual confounding cannot be excluded.

Whilst the sample size and research design were major strengths of this study, there were some drawbacks that should to be considered. The sample of children with ASD was based on parental report, which was not independently validated by the research team. The analyses were repeated to only include those children who had been reported as having ASD at all three ages (5, 7, and 11 years) and the effects remained and were stronger (further details available upon request from the corresponding author). Given that children with ASD have poorer reading and literacy skills compared to children without ASD, the self-report method may have introduced an additional source of error. It may also be the case that children with ASD at the lowest level of functioning were not able to complete the questionnaires and so dropped out of the study. Population and sample weights were used to minimise such unequal attrition. Additionally, self-report is arguably a more accurate way to measure sibling bullying, as parents are not aware of all the conflicts that happen between siblings with much of the bullying occurring behind closed doors. Therefore, the alternative method of measuring sibling bullying, would introduce different measurement error. However, it should be noted that there was no independent corroboration of the sibling bullying involvement, e.g. by the sibling. The measure of sibling bullying perpetration and victimisation are both based on single items and future studies should attempt to utilise multi-item scales. Furthermore, as the ASD diagnosis status of siblings was not determined, this, again, may have introduced an additional source of error. Similarly, because ethnicity was defined using binary categorisation, it may be that heterogeneity that exists within ethnic group was masked (Tippett et al. 2013). Given the very large sample size, this additional error was likely minimal but nonetheless the findings should be interpreted with this in mind. Finally, associations between sibling bullying and internalizing and externalizing behaviour were assessed concurrently. Thus causality cannot be inferred. However, there is currently only one prospective study of sibling victimization and its adverse effects that found that sibling victimization is a temporal precursor of later internalizing problems and a dose-response effect suggests a potentially causal effect of sibling victimization (Bowes et al. 2014).

## Conclusions

In this population-based sample of children with and without ASD, it was found that children with ASD were more likely than those without ASD to be victims and perpetrators of sibling bullying as opposed to only victims or only perpetrators. Sibling bullying was associated with more internalizing and externalizing problems irrespective of ASD status.

These findings have important implications for the provision of resources for children with ASD. Given that the evidence for peer bullying in children with ASD is well established, resources are already being targeted to improve peer relations in children with ASD. Our novel evidence suggests additional targeting of resources at improving interactions between children with ASD and their siblings. Given, that our findings show that the bullying is more likely to be two-way in children with ASD compared to those without ASD, interventions should focus on the siblings as well as the affected child. Overall, a reduction in sibling bullying is likely to reduce social and emotional adversities in both those with ASD and without ASD. Such interventions would reduce the economic and social burden of adverse psychopathology associated with sibling bullying.

## References

[CR1] American Psychiatric Association (2013). Diagnostic and statistical manual of mental disorders.

[CR2] Bagenholm A, Gillberg C (1991). Psychosocial effects on siblings of children with autism and mental-retardation—A population-based study. Journal of Mental Deficiency Research.

[CR3] Baird G, Simonoff E, Pickles A, Chandler S, Loucas T, Meldrum D, Charman T (2006). Prevalence of disorders of the autism spectrum in a population cohort of children in South Thames: the Special Needs and Autism Project (SNAP). Lancet.

[CR4] Bowes L, Wolke D, Joinson C, Lereya ST, Lewis G (2014). Sibling bullying and risk of depression, anxiety, and self-harm: A prospective cohort study. Pediatrics.

[CR5] Brown JR, DonelanMcCall N, Dunn J (1996). Why talk about mental states? The significance of children’s conversations with friends, siblings, and mothers. Child Development.

[CR6] Cappadocia MC, Weiss JA, Pepler D (2012). Bullying experiences among children and youth with autism spectrum disorders. Journal of Autism and Developmental Disorders.

[CR7] Connelly R, Platt L (2014). Cohort profile: UK Millennium Cohort Study (MCS). International Journal of Epidemiology.

[CR8] Constantino JN, Lajonchere C, Lutz M, Gray T, Abbacchi A, McKenna K (2006). Autistic social impairment in the siblings of children with pervasive developmental disorders. American Journal of Psychiatry.

[CR9] Cowie H, Sharp S, Smith PK, Sharp S (1994). Empowering pupils to take positive action against bullying. School bullying: Insights and perspectives.

[CR10] Dawson G, Estes A, Munson J, Schellenberg G, Bernier R, Abbott R (2007). Quantitative assessment of autism symptom-related traits in probands and parents: Broader Phenotype Autism Symptom Scale. Journal of Autism and Developmental Disorders.

[CR11] Dillenburger K, Jordan JA, McKerr L, Keenan M (2015). The millennium child with autism: Early childhood trajectories for health, education and economic wellbeing. Developmental Neurorehabilitation.

[CR12] Downey DB, Condron DJ (2004). Playing well with others in kindergarten: The benefit of siblings at home. Journal of Marriage and Family.

[CR13] Duncan RD (1999). Peer and sibling aggression: An investigation of intra- and extra-familial bullying. Journal of Interpersonal Violence.

[CR14] Elliot CD, Smith P, McCulloch K (1996). British ability scales second edition (BAS II). Administration and scoring manual.

[CR15] Goodman R (1997). The strengths and difficulties questionnaire: A research note. Journal of Child Psychology and Psychiatry.

[CR16] Hagenaars, A., de Vos, K., & Zaida, M. A. (1994). Poverty statistics in the late 1980s: Research based on micro-data. Retrieved from Luxembourg.

[CR17] Hebron J, Humphrey N, Oldfield J (2015). Vulnerability to bullying of children with autism spectrum conditions in mainstream education: a multi-informant qualitative exploration. Journal of Research in Special Educational Needs.

[CR18] Kaartinen M, Puura K, Helminen M, Salmelin R, Pelkonen E, Juujarvi P (2014). Reactive aggression among children with and without autism spectrum disorder. Journal of Autism and Developmental Disorders.

[CR19] Kaminsky L, Dewey D (2001). Siblings relationships of children with autism. Journal of Autism and Developmental Disorders.

[CR20] Little L (2002). Middle-class mothers’ perceptions of peer and sibling victimization among children with Asperger’s syndrome and nonverbal learning disorders. Issues Compr Pediatr Nurs.

[CR21] Matson JL, Nebel-Schwalm MS (2007). Comorbid psychopathology with autism spectrum disorder in children: An overview. Research in Developmental Disabilities.

[CR22] McHale SM, Crouter AC, McGuire SA, Updegraff KA (1995). Congruence between mothers and fathers differential treatment of siblings—Links with family-relations and childrens well-being. Child Development.

[CR23] Meadan H, Stoner JB, Angell ME (2010). Review of literature related to the social, emotional, and behavioral adjustment of siblings of individuals with autism spectrum disorder. Journal of Developmental and Physical Disabilities.

[CR24] Pickles A, Durkin K, Mok PLH, Toseeb U, Conti-Ramsden G (2016). Conduct problems co-occur with hyperactivity in children with language impairment: A longitudinal study from childhood to adolescence. Autism & Developmental Language Impairments.

[CR25] Robbins TW, James M, Owen AM, Sahakian BJ, McInnes L, Rabbitt P (1994). Cambridge Neuropsychological Test Automated Battery (CANTAB): A factor analytic study of a large sample of normal elderly volunteers. Dementia.

[CR26] Ross P, Cuskelly M (2006). Adjustment, sibling problems and coping strategies of brothers and sisters of children with autistic spectrum disorder. Journal of Intellectual & Developmental Disability.

[CR27] Russell G, Rodgers LR, Ford T (2013). The strengths and difficulties questionnaire as a predictor of parent-reported diagnosis of autism spectrum disorder and attention deficit hyperactivity disorder. PLoS ONE.

[CR28] StataCorp. (2015). Stata statistical software: Release 14. Retrieved from College Station, Texas.

[CR29] Sterzing PR, Shattuck PT, Narendorf SC, Wagner M, Cooper BP (2012). Bullying involvement and autism spectrum disorders prevalence and correlates of bullying involvement among adolescents with an autism spectrum disorder. Archives of Pediatrics & Adolescent Medicine.

[CR30] Stoner JB, Angell ME, House JJ, Bock SJ (2007). Transitions: Perspectives from parents of young children with autism spectrum disorder (ASD). Journal of Developmental and Physical Disabilities.

[CR31] Stormshak EA, Bellanti CJ, Bierman KL (1996). The quality of sibling relationships and the development of social competence and behavioral control in aggressive children. Developmental Psychology.

[CR32] Tippett N, Wolke D (2015). Aggression between siblings: Associations with the home environment and peer bullying. Aggressive Behavior.

[CR33] Tippett N, Wolke D, Platt L (2013). Ethnicity and bullying involvement in a national UK youth sample. Journal of Adolescence.

[CR34] Toseeb U, Pickles A, Durkin K, Botting N, Conti-Ramsden G (2017). Prosociality from early adolescence to young adulthood: A longitudinal study of individuals with a history of language impairment. Research in Developmental Disabilities.

[CR35] Toth K, Dawson G, Meltzoff AN, Greenson J, Fein D (2007). Early social, imitation, play, and language abilities of young non-autistic siblings of children with autism. Journal of Autism and Developmental Disorders.

[CR36] Tucker CJ, Finkelhor D, Shattuck AM, Turner H (2013). Prevalence and correlates of sibling victimization types. Child Abuse & Neglect.

[CR37] Tucker CJ, Finkelhor D, Turner H, Shattuck AM (2014). Family dynamics and young children’s sibling victimization. Journal of Family Psychology.

[CR38] Tucker CJ, Finkelhor D, Turner H, Shattuck AM (2014). Sibling and peer victimization in childhood and adolescence. Child Abuse & Neglect.

[CR39] Updegraff KA, Thayer SM, Whiteman SD, Denning DJ, McHale SM (2005). Relational aggression in adolescents’ sibling relationships: Links to sibling and parent-adolescent relationship quality. Family Relations.

[CR40] van Roekel E, Scholte RHJ, Didden R (2010). Bullying among adolescents with autism spectrum disorders: Prevalence and perception. Journal of Autism and Developmental Disorders.

[CR41] van Steensel FJA, Bogels SM, Perrin S (2011). Anxiety disorders in children and adolescents with autistic spectrum disorders: A meta-analysis. Clinical Child and Family Psychology Review.

[CR42] Verte S, Roeyers H, Buysse A (2003). Behavioural problems, social competence and self-concept in siblings of children with autism. Child Care Health and Development.

[CR43] Volk AA, Camilleri JA, Dane AV, Marini ZA (2012). Is adolescent bullying an evolutionary adaptation?. Aggressive Behavior.

[CR44] Wolke D, Samara MM (2004). Bullied by siblings: association with peer victimisation and behaviour problems in Israeli lower secondary school children. Journal of Child Psychology and Psychiatry.

[CR45] Wolke, D., & Skew, A. J. (2011). Bullied at home and at school: Relationship to behaviour problems and unhappiness. Retrieved from London.

[CR46] Wolke D, Tippett N, Dantchev S (2015). Bullying in the family: Sibling bullying. Lancet Psychiatry.

